# Use of Teduglutide in Children With Intestinal Failure: A Systematic Review

**DOI:** 10.3389/fnut.2022.866518

**Published:** 2022-06-14

**Authors:** Francesca Gigola, Maria Chiara Cianci, Roberto Cirocchi, Maria Chiara Ranucci, Marco Del Riccio, Riccardo Coletta, Antonino Morabito

**Affiliations:** ^1^Department of Pediatric Surgery, Meyer Children’s Hospital Academic Centre, Florence, Italy; ^2^Department of Medicine and Surgery, University of Perugia, Perugia, Italy; ^3^Department of Digestive and Emergency Surgery, Santa Maria di Terni Hospital, University of Perugia, Perugia, Italy; ^4^Postgraduate School of Hygiene and Preventive Medicine, Florence, Italy; ^5^School of Health and Society, University of Salford, Salford, United Kingdom; ^6^Meyer Children’s Hospital, Department of Neurofarba, University of Florence, Florence, Italy

**Keywords:** glucagon-like peptide 2, rare disease, parenteral nutrition, short bowel syndrome, malabsorption, intestinal adaptation

## Abstract

**Background and Objectives:**

Short-bowel syndrome (SBS) results from the loss of a significant portion of the small intestine leading to a state of malabsorption. After an intestinal loss, there is a process of adaptation involving the Glucagon-Like Peptide-2 (GLP-2), an enteroendocrine peptide also involved in nutrient absorption. Teduglutide is a recombinant analog of GLP-2 approved in 2016 to treat selected SBS pediatric patients who are dependent on parenteral support. The present systematic review aims to evaluate the efficacy of Teduglutide in pediatric patients with SBS in reducing the need for parenteral nutrition (PN).

**Materials and Methods:**

We performed a literature search on MEDLINE and Embase to include articles up to November 2021. We included articles that involved using Teduglutide in the SBS pediatric population to define its efficacy in reducing the need for PN. The key words used were GLP-2, teduglutide, child.

**Results:**

Fourteen studies completely fulfilled the inclusion criteria. Two hundred 23 patients were treated with Teduglutide, and the median duration of treatment was 45 weeks (IQR: 36–52.5 weeks). One-hundred and fifty-two patients were treated with 0.05 mg/Kg/d of subcutaneous Teduglutide, 38 received 0.025 mg/Kg/d and 8 received either 0.125 mg/Kg/d or 0.20 mg/Kg/d. A total of 36 patients achieved enteral autonomy (EA) after a median of 24 weeks of treatment (IQR: 24–48 weeks) and 149 patients showed a reduction in PN needs in terms of volume, calories, or hours per day. Eleven studies reported complications: gastrointestinal were the most common, with 89 cases reported in treated patients and 11 in non-treated patients.

**Conclusion:**

Teduglutide appears safe and effective in reducing PN requirements and improving EA in the pediatric population. However, more studies are needed to understand its efficacy in the long term and after discontinuation and possible complications.

**Systematic Review Registration:**

[https://www.crd.york.ac.uk/prospero/], identifier [CRD42022301593].

## Introduction

Intestinal Failure (IF) is caused by loss of a significant portion of the small intestine following congenital disease, acquired causes or surgical resection. With the inability to maintain protein, electrolyte, and micronutrient balance due to malabsorption the body may fail to sustain growth in affected children.

Short Bowel Syndrome (SBS) is a complex condition that requires specialized care to prevent complications: for these reasons, treatment should be carried out by multidisciplinary intestinal rehabilitation teams. Such a team will help combine medical approaches and surgical techniques to achieve enteral autonomy (EA) and prevent IF-associated complications. SBS patients require PN to get nutrients and fluids that cannot be obtained through enteral nutrition. It is of the utmost importance to find ways to help them achieve EA and weaning from PN since this will improve their overall quality of life and psychological status (1). Moreover, it will reduce the risk of complications related to vascular access and hepatobiliary damage associated with PN dependency. There are different surgical techniques that can be used to treat SBS patients to help them achieve EA, but sometimes surgery is not an available option, and it is important to find other ways to achieve EA in these patients.

After a significant intestinal loss, the remaining intestine will undergo adaptation. Gradually, changes will take place to maximize the absorptive area and provide the body with an adequate quantity of fluids, macro-, and micronutrients. This event is particularly true in pediatric patients, especially under 5 years of age, because bowel length increases in the first 5 years of life (2, 3). In the first months after an intestinal loss patients will suffer diarrhea with significant fluid loss. Crypts will deepen after some time, and villi will become hypertrophic, muscle layers will thicken, granting better fluids and nutrient absorption (2, 4). This process is also known as bowel adaptation (5) and while it takes some time to complete, it is usually associated with bowel dilatation, which worsens intestinal peristalsis because muscle contractions are less effective in a dilated segment (6). In SBS patients some intestinal sections are characterized by reduction of peristaltic movements due to dilatation, creating an ideal environment for bacterial proliferation and overgrowth. This alteration of intestinal microbiota is associated with abdominal pain, inflammation and damage of the mucosa, generation of toxic products such as D-Lactic acid and bacterial translocation with potential risk for sepsis (6).

The adaptation process involves several hormones, such as endothelial growth factor (EGF), growth hormone (GH), and Glucagon-like peptide-2 (GLP-2); among these, GLP-2 is a crucial component. GLP-2 is a 33-amino-acid peptide derived from a proglucagon precursor that also carries the sequence of glucagon and GLP-1. This peptide is secreted by enteroendocrine L cells, which can be found in the basal aspect of the intestinal mucosa in the distal ileum and proximal colon; nutrients induce its secretion in the intestinal lumen and then the peptide acts through second effectors such as IGF-1. GLP-2 improves nutrient absorption and gut-barrier function and slows motility in the short term. Thus, it promotes contact of nutrients with the intestinal mucosa, and it may also act as a regulator of blood supply to the gut (7). GLP-2 acts as a trophic factor for the small bowel mucosa (2, 8, 9), increasing the absorptive capacity of the intestine after intestinal loss and promoting adaptation (9, 10). Unfortunately, human GLP-2 is deactivated by dipeptidyl peptidase-IV, and for this reason, it has a half-life of 7 min if administered subcutaneously to humans (10).

For this reason, researchers studied the formation of an analog recombinant form of this peptide with a longer lifespan, Teduglutide. In 2016 the European Medicines Agency approved the use of Teduglutide in pediatric patients (ages 1–17 years old); Teduglutide is resistant to the action of dipeptidyl peptidase IV, and it has a longer half-life compared to human GLP-2 (11, 12). After its approval, many attempts have been made to use it in the pediatric population; we conducted a systematic review of the articles that assessed the effectiveness of Teduglutide in reducing the need for PN or even achieving EA in children affected by IF.

## Materials and Methods

We conducted a literature search using MEDLINE and Embase to include articles up to November 2021 using the following keywords: glucagon-like peptide, teduglutide, child. We included studies with an English abstract available.

Inclusion criteria for this systematic review were original articles including pediatric patients (aged < 18 years old) affected by SBS and IF and dependent on PN, treated with a recombinant analog of GLP-2 Teduglutide. The primary endpoint was the efficacy of Teduglutide in reducing the dependence of PN. Secondary endpoints included evaluating plasmatic citrulline levels and stool frequency and consistency improvement.

The search strategy was done according to the PRISMA statement, and the present systematic review analysis was registered on the PROSPERO database (registration number: CRD42022301593) (13).

After excluding duplicates, two authors (F.G. and MC.C) reviewed the articles independently and in a blind manner and articles were first screened for inclusion by title and abstract. Any disagreement was resolved by consensus.

When two articles were published by the same research group or in case of potential patients’ overlap, we considered the study that included a higher number of patients to avoid duplication of data.

Data were extracted using an internal spreadsheet, and the following information was extracted: (1) Study characteristics; title, first author, year of publication, country of the hospital in which the study was conducted, study design, number of patients enrolled, and number of patients treated; (2) characteristics of participants and treatment; (3) outcome of treatment; (4) complications.

Given the paucity of available studies and patients identified with our selection criteria, data are reported as a narrative review.

Quality assessment of studies was performed independently and in a blind manner by two authors, according to The Risk of Bias in Non-randomized Studies-of-Intervention (ROBINS-I) tool available at https://methods.cochrane.org/methods-cochrane/robins-i-tool (14). Any disagreement was resolved by consensus.

## Results

**FIGURE 1:**
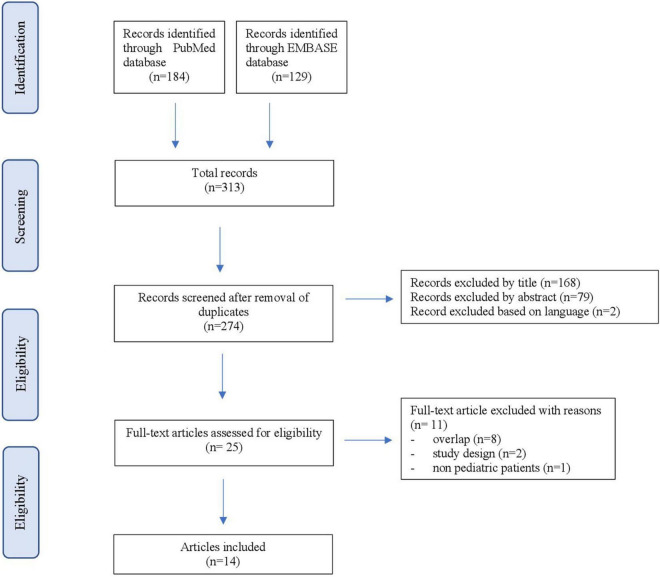
PRISMA flowchart.

The literature search identified 313 studies (184 from MEDLINE, 129 from EMBASE). After removing 39 duplicates, 274 studies were screened for inclusion based on title and abstract review. Given the lack of records and the condition’s rarity, we considered eligible abstracts and posters when they included relevant information regarding treatment efficacy and fulfilled inclusion criteria. The abstract or the complete text, when available, were then examined in detail. Fourteen studies fulfilled the inclusion criteria and were finally included in our review (9, 12, 15–26). Principal reasons for exclusion were overlapped with published studies (*n* = 8), study design (*n* = 2) and inclusion of non-pediatric patients (*n* = 1). The complete screening process is detailed in Figure 1.

The main characteristics of those studies are summarized in Table 1.

**TABLE 1:** Characteristics of included studies.

References	Center	Type of study	Registration	Duration of treatment	Number of patients enrolled	Number of patients underwent teduglutide
Carter et al. (18)	Multicenter study at 17 sites in the US and the United Kingdom	Prospective	ClinicalTrials.gov: NCT01952080	12 weeks	42	37
Busoni et al. (19)	One reference IF center in Latin America	Retrospective		22 months	3	3
Ferreiro et al. (20)	Single center in Spain	Case report		60 weeks	1	1
Hill et al. (22)	U	Prospective	ClinicalTrials.gov: NCT02949362	6 months	16	16
Kinberg (23)	Single center in US	Retrospective		10 months (3–18)^x^	8	8
Lambe (25)	Single center in France	Prospective	ClinicalTrials.gov: NCT03562130	48 weeks	25	25
Kocoshis et al. (24)	Multicenter in 24 centers in North America and Europe	Prospective	ClinicalTrials.gov, NCT02682381	24 weeks	59	50
Martìnez et al. (26)	Single center in Argentina	Retrospective		42 weeks ± 42.5 weeks^y^	4	4
Ramos-Boluda et al. (16)	Multicenter study at 8 sites in Spain	Prospective		12 months	17	17
Ribeiro-Mourão et al. (15)	Single center in Portugal	Prospective		6 months	4	4
Rumbo et al. (17)	Single center in Argentina	Case report		50 weeks	1	1
Sigalet et al. (9)	Two sites in Canada	Prospective	ClinicalTrials.gov: NCT01573286	42 days	7	7
Sigalet et al. (12)	Three sites in Canada	Prospective	ClinicalTrials.gov: NCT01573286	42 days	6	6
Mercer et al. (21)	U	Prospective	ClinicalTrials.gov: NCT02954458	6 months	55	44
Total					248	223

*U, unknown; ^x^Median duration and range, ^y^Mean duration and standard deviation.*

The 14 selected studies included 248 patients, of which 223 were treated with Teduglutide. Duration of treatment went from 42 days to 60 weeks with a median of 45 weeks (IQR: 36–52.5 weeks). Seven studies were single center studies, while five were multicentric. Two papers did not report the country or the center of the study. Nine were prospective, three were retrospective analyses, and two were case reports in the included studies.

All but one study included patients aged 1–17; one study included children < 1 year of age (9). The age range went from 1.5 months to 17 years. Three studies did not specify the age range. All patients were affected by SBS-IF; specific inclusion criteria of each study are reported in Table 2.

**TABLE 2:** Characteristics of patients.

References	Age	Sex, n (%)	Characteristics of patients		
Carter et al. (18)	3 years (1–14)^x^	*M*: 28 (67%) *F*: 14 (33%)	Aged 1–17 years	SBS-IF	≥ 12-month history of SBS and dependence on PN (defined as PN and/or intravenous fluids) for at least 30% of caloric and/or fluid/electrolyte needs. PN needs were required to be stable at baseline, without any clinically meaningful or substantial reduction in PN or advancement in enteral nutrition (EN; oral and/or tube feeding) for ≥3 months.
Busoni et al. (19)	9,7 years (9–10)^y^	*M*: 3 (100%) *F*: 0 (0%)	Aged 1–17 years	SBS-IF	No changes in nutritional support in the previous 3 months
Ferreiro et al. (20)	7 years	*M*: 0 (0%) *F*: 1 (100%)			Premature (33 weeks)
Hill et al. (22)	U	U	Aged 1–17 years	SBS-IF	Patients who completed the core phase III TED-C13-003 12-week study (NCT01952080; EudraCT 2013-004588-30) were eligible. All patients who enrolled had received TED 0.0125, 0.025, or 0.05 mg/kg once daily in the phase III study. Patients had a 2.4- to 3.3-year gap after the 12-week study and enrolment in this study. Treatment with TED was provided if their parenteral support plateaued or deteriorated after prior TED treatment ended.
Kinberg (23)	5 years (1–16)^x^	*M*: 4 (50%) *F*: 4 (50%)	Aged 1–17 years	SBS-IF	
Lambe (25)	10 years (5–16)^y^	*U*	Aged 1–17 years	SBS-IF	Patients followed in the authors’ center with > 2 years on Home PN, small bowel length < 80cm and who had reached a plateau on long-term PN (no decrease of PN in the previous 6 months)
Kocoshis et al. (24)^z^	SOC 6 years ± 5 0.025 7 years ± 4 0.05 6 years ± 4	*M*: 41 (70%) *F*: 18 (30%)	Aged 1–17 years	SBS-IF	≥ 12-month history of SBS and dependence on PN (defined as PN and/or intravenous fluids) for at least 30% of caloric and/or fluid/electrolyte needs. PN needs were required to be stable at baseline, without any clinically meaningful or substantial reduction in PN or advancement in enteral nutrition (EN; oral and/or tube feeding) for ≥3 months.
Martìnez et al. (26) ^x^	12 years (6–17)	*M*: 3 (75%) *F*: 1 (25%)	Aged 1–17 years	SBS-IF	
Ramos-Boluda et al. (16)^y^	68 months (12–121)	U	Aged 1–17 years	SBS-IF	Remnant bowel less of 150 cm, dependent on PN, and with no surgical interventions or changes in PN in the previous 3 months.
Ribeiro-Mourão et al. (15)^y^	9 years (6–12)	*M*: 1 (25%) *F*: 3 (75%)	Aged 1–17 years	SBS-IF	Dependent on PN, no changes in composition of PN in the previous 6 months
Rumbo et al. (17)	6 years	*M*: 1 (100%) *F*: 0 (0%)			
Sigalet et al. (9)	5.4 months ± 3.2^z^	U	Aged < 1 year	SBS-IF	Requirement for > 50% of calories by PN, more than 45 days from last intestinal surgery OR Intestinal Failure with a requirement for > 50% of calories by PN, more than 45 days from an intestinal resection, independent of the length of remnant small intestine OR Gastroschisis with a requirement for > 50% of calories by PN, and more than 45 days from last abdominal/intestinal surgery
Sigalet et al. (12)	U	U	Aged 1–17 years	SBS-IF	Anatomic SBS, with < 40% of expected bowel length and a requirement for > 30% of calories by PN, > 3 months (90 days) from last intestinal surgery; or gastroschisis, with a requirement for > 30% of calories by PN and > 3 months from last intestinal surgery
Mercer et al. (21)	U	U	Aged 1–17 years	SBS-IF	Patients who completed a core phase III TED-C14-006 24-week study (NCT02682381 EudraCT 2015-002252-27), were eligible

*SBS, Short Bowel Syndrome; IF, Intestinal Failure; PN, Parenteral Nutrition; EN, Enteral Nutrition.*

*^x^Median (range), ^y^Mean (range), ^z^Mean ± Standard Deviation.*

Gender was specified in 8 studies, out of 122 patients for which it was known 81 (67%) were male, and 41 (33%) were female.

Ten studies specified the underlying diagnosis that led to SBS-IF, with a total number of 144 patients, while four studies did not mention the primary diagnosis. The most common diagnosis was gastroschisis (*n* = 44, 31%), followed by volvulus (*n* = 41, 28%), necrotizing enterocolitis (*n* = 36, 25%) and intestinal atresia (*n* = 23, 16%). The causes of resection are summarized in Table 3.

**TABLE 3:** Causes of intestinal resection.

References	Necrotizing enterocolitis	Midgut volvulus	Intestinal atresia	Gastroschisis	Other (or non-specified)
Carter et al. (18)	8	15	8	12	3
Busoni et al. (19)					3 SBS type 2
Ferreiro et al. (20)	1				
Hill et al. (22)	U	U	U	U	U
Kinberg (23)	5			2	1
Lambe (25)	U	U	U	U	25 (6 SBS type 1, 11SBS type 2, 8 SBS type 3)
Kocoshis et al. (24)	10	19	3	22	5
Martìnez et al. (26)		1	2	1	1
Ramos-Boluda et al. (16)	6	3	3	2	3 (2 Hirschsprung, 1 PIPO)
Ribeiro-Mourão et al. (15)		2	1	1	
Rumbo et al. (17)			1		
Sigalet et al. (9)	3	1	2	1	1
Sigalet et al. (12)	3		3	3	1
Mercer et al. (21)	U	U	U	U	U
Total	36	41	23	44	43

*ASBS, Short Bowel Syndrome; PIPO, Pediatric Intestinal Pseudo-Obstruction. Some patients were affected by more than one of these conditions, the total number does not always add up to the total of patients.*

**FIGURE 2:**
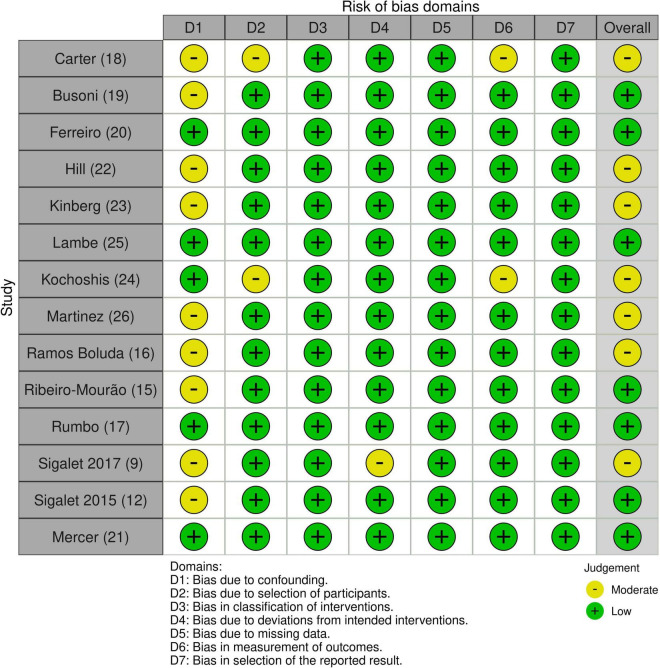
Quality assessment results according to ROBINS-I.

Figure 2 shows the quality assessment results conducted using the ROBINS-I tool for non-Randomized studies of Intervention (14). All studies turned out to be at low risk regarding bias in classification of interventions, selection of reported results and discrimination due to missing data. Two reports (18, 24) were at moderate risk regarding the selection bias due to the allocation of open-label treatment and the resulting remote-control cohort. This caused patients selection to be influenced by post-intervention variables; furthermore, the sample size was based on the available patient population rather than a statistical power calculation. Moreover, Kocoshis et al. (24) excluded patients who were considered incapable of advancing in enteral nutrition, and the authors did not define how patients were considered as such. Carter et al.’s (18) analysis presented a small control cohort of a younger population that may be more capable of endogenous intestinal adaptation. Bias due to deviations from intended interventions was assessed as low for all the studies except for Sigalet et al. (9) (moderate): two patients did not adhere to intervention due to adverse events (which were non-Teduglutide related) and because the trial was discontinued early after a drop in potency.

Regarding bias in measuring outcomes, all but two studies were considered at low risk, while Carter et al.’s and Kocoshis et al. studies turned out at moderate risk (18, 24). In these two studies, assessment methods are comparable across groups. Still, outcome measures could have been influenced by knowledge of the intervention: a non-blinded control sample coupled with expected clinical benefit from teduglutide may have biased parenteral support adjustments.

Bias due to confounding is low for five studies out of 14 (17, 20, 21, 24, 25). At the same time, the remainders present a moderate risk of bias due to small sample size and short follow up (9, 12, 15, 18, 19, 26), different periods of treatment with teduglutide (26) and absence of stratification of results based on age bowel length (16, 23) and teduglutide dose (22).

In conclusion, the overall risk of bias is considered low for seven out of fourteen studies (12, 15, 17, 19–21, 25), while it is classified as moderate for seven surveys (9, 16, 18, 22–24, 26).

Intestinal length varied from a minimum of 0 cm to a maximum of 175 cm (18, 20). Three papers did not specify the intestinal length at the beginning of treatment; the intestinal length mainly was determined by surgery with a total of 43 cases, even though only five papers stated the method used to determine the length of the remaining intestine in patients.

Eight studies evaluated the presence of the ileocecal valve. In 38 patients the Ileocecal valve was preserved (28%), while in 98 patients it was absent (72%) (12, 15–18, 23, 24, 26). In addition, in 6 studies, patients had been submitted to prior lengthening surgery (9, 15, 17–20) as the STEP procedure (serial transverse enteroplasty) in 82.4% of cases (15, 17, 18, 20).

Length of the colon was reported in 8 papers, mainly as a percentage of the remaining colon, ranging from 8% of total length to the presence of the whole colon.

Of the 223 treated patients 152 (61%) were treated with 0.05 mg/Kg/d of subcutaneous Teduglutide, 38 (16%) received 0.025 mg/Kg/d, 8 (3%) received 0.125 mg/Kg/d and 8 (3%) were treated with 0.20 mg/Kg/d. In 17 patients (7%) the dose was not specified. Twenty-five patients (10%) were in the Standard of Care (SOC) arm and did not receive Teduglutide. Characteristics of Teduglutide administration are summarized in Table 4.

**TABLE 4:** Characteristics of teduglutide administration.

References	Dose, *n* (%)					
	SOC	0.020 mg/kg/day	0.125 mg/kg/day	0.025 mg/kg/day	0.05 mg/kg/day	Unknown
Carter et al. (18)	5 (12%)		8 (19%)	14 (33%)	15 (36%)	
Busoni et al. (19)					3 (100%)	
Ferreiro et al. (20)					1 (100%)	
Hill et al. (22)					16 (100%)	
Kinberg (23)						8 (100%)
Lambe (25)					25 (100%)	
Kocoshis et al. (24)	9 (15%)			24 (41%)	26 (44%)	
Martìnez et al. (26)					4 (100%)	
Ramos-Boluda et al. (16)					17 (100%)	
Ribeiro-Mourão et al. (15)						4 (100%)
Rumbo et al. (17)					1 (100%)	
Sigalet et al. (9)		1 (17%)				5 (83%)
Sigalet et al. (12)		7 (100%)				
Mercer et al. (21)	11 (20%)				44 (80%)	
Total	25 (10%)	8 (3%)	8 (3%)	38 (16%)	152 (61%)	17 (7%)

*SOC, Standard of care.*

After treatment, a total of 36 patients (16%) achieved EA after a median of 24 weeks of treatment (IQR: 24–48 weeks), and 149 patients (67%) showed a reduction in PN needs in terms of volume, calories, or hours per day. Thirty-eight patients who reduced PN requirements were in the 0.025 mg/kg/day group, 105 were in the 0,05 mg/kg/day group, one patient was in the 0.02 mg/kg/day group, and five patients the dose was not known. Study results are reported in Table 5.

**TABLE 5:** Results.

References	PN before treatment	EN before treatment	PN after treatment	Weaned	PN after discontinuation	EN after discontinuation	Weight after	Height after	Stool improvement	Citrulline levels
Carter et al. (18)^x^	U	29 (69%)	SOC: −0 (−0.3, 1.4) L/week, −0 (−2.0, 0.6) hours per day, −1 (−5, 5) kcal/day/kg		After 4 weeks of suspension, 2 patients resumed PN	SOC	U	U	U	U
			0.0125: −0 (−2.5, 0) L/week, −0 (0,2.0) hours per day, −2 (−12, 3) kcal/day/kg			0.0125: +1,1 (0–12.5) L/week				0.0125: + 1 μmol/L (−0.8, 22.9)
			0.025: −2,3 L/week (−6.9−0), −4 (−9.0−2.0) hours per day, −17 (−39, 2) kcal/day/kg	1 at week 11		0.025: +2,3 (−0.9–8.8) L/week				0.025: + 5.4 μmol/L (1.1, 17.2)
			0.05: −1,3 L/week (−11 −1) at 12 weeks, −3 h per day (−12.0−0.8), −17 (−45, 53) kcal/day/kg	3 at week 4, 8, 12		0.05: +0.7 (0–3.9) L/week				0.05: +7.5 μmol/L (−13.9, 56.5)
Busoni et al. (19)^y^	3,000 ml/week	U	1 patient: −100% at 10 weeks	1 at week 10	U	U	+1,8 kg (1.3–2)	+5,7 cm (4,5–6,5)	Yes	U
	7,000 ml/week		1 patient: −30% at 4 weeks, −71% at 8 weeks (reduced infusions at 5 nights)							
	12,600 ml/week		1 patient: −28% at 45 weeks							
Ferreiro et al. (20)	16,100 ml/week, 25 Kcal/kg	U	0 ml/day at 30 weeks	1 at week 30	U	U	+4,4 kg	+11 cm	Yes	Increased
Hill et al. (22)^z^	U	U	−3.7 ± −15.72 kcal/kg/day at Cycle 1Day1 (*n* = 15) and −21.6 ± 17.90 kcal/kg/day at Cycle 1 Week 24 (*n* = 13). Number of days per week: −0.4 ± 1.92 days per week at C1D1 (*n* = 14) and −0.9 ± 2.23 days per week at C1W24 (*n* = 12)	0	U	U	Stable	Stable	U	U
Kinberg (23)^x^	U	U	Six patients: −1.8 (0–2.4) L/week One patient reduced number of hours per day	0	U	U	U	U	U	U
Lambe (25)^z^	NPEI/REE index was 95 ± 28%	U	At 12 weeks: all patients > 20% decrease of PN requirements (−33%, NPEI/REE index 64 ± 22%) At 24 weeks: mean NPEI/REE index 57.5 ± 25%	8 at 48 weeks	U	U	Stable	Stable	Y	15.2 ± 9 mol/l (mean) at baseline to 26.5 ± 21.6 mol/l at week 12, 31.6 ± 21.6 mol/l at week 24 and 36 mol/l ± 25.2 at week 48
Kocoshis et al. (24)^z^	**SOC:** 79.6 ± 31.12 mL/kg/d, 44.6 ± 22.53 kcal/kg/d, 6.6 ± 1.33 days per week, 12.6 ± 5.50 h per day	52 (88%)	**SOC:** > 20% reduction in 1 patient at week 24, –6.0 ± 4.55 mL/kg/d, −0.5 ± 4.95 kcal/kg/d, −0 days per week, –0.2 ± 0.69 h per day	0	**SOC:** stable	Still reduced	**U**	**SOC:** height z-score change from baseline: −0.23 +/−0.26	U	Baseline SOC: 12.6+/−8.43 (8 patients) 0.025: 17.9 ±12.64 (21 pts) 0.05: 16.0 + −11.54 (24 pts) Week 24 SOC: 12.3 + −6.57 0.025: 25.5 + −15.90 0.05: 29.0 + −15.23
	**0.025:** 56.8 ± 25.24 ml/kg/day, 43.3 ± 21.10 kcal/kg/d, 6.5 ± 1.10 days per week, 11.7 ± 3.03 h per day		**0.025:** > 20% reduction in 13 patients at week 24, –16.2 ± 10.52 mL/kg/d, –14.9 ± 8.29 kcal/kg/d, –0.9 ± 1.78 days per week, –2.5 ± 2.73 h per day		**0.025:** increased in 23 patients, + 76.9% ± 117.19% in volume, +82.7% ± 136.27% in calories			**0.025:** height z-score change from baseline: −0.09± 0.3		
	**0.05:** 60 ± 29.19 ml/kg/day, 43.3 ± 16.52 kcal/kg/d, 6.6 ± 0.79 days per week, 11.2 ± 2.99 h per day		**0.05:** > 20% reduction in 18 patients at week 24, –23.3 ± 17.50 mL/kg/d, –19.0 ± 14.28 kcal/kg/d, –1.3 ± 2.24 days per week, –3.0 ± 3.84 h per day		**0.05**: increased in 26 patients, + 79.5% ± 134.49% in volume, + 86.47% ± 128.11% in calories			**0.05**: height z-score change from baseline: −0.04 ± 0.24		
Martìnez et al. (26)^z^	11.55 ± 3.5 L/week	15% ± 8.8% (of total intake)	1: 12.9 L/week at week 14 1: 11.7 L/week at week 8	2 at weeks 44, 101	U	U	U	U	U	U
Ramos-Boluda et al. (16)^y^	55 ml/kg/d (8–210) 33 kcal/kg/d (0–65) 2 patients only IV fluids	U	4 decreased PN requirements 1 No change 1 discontinuation Response rate (< 20% of PN requirements) of 47% at month 3, 87% at 6 months, and 93% at 1 year.	3 at month 3 4 at month 6 3 at month12	U	U	U	U	Y	Baseline: 20 mmol/l (7.8 −51) 12 months: 37.9 mmol/l (9–67)
Ribeiro-Mourão et al. (15)	3 patients: 145% PN/REE, 1 patient 97% PN/REE;	U	1 month: reduction of at least 1 day/week (all patients) 6 months: 1 patient 2 days reduction (1 patient discontinued after 1 month)	2 at 6 months	U	Increased by 10% at month 1, 73% at month 6	+ 1.3 kg at month 1 + 2.8 kg at month 6	+1.6 cm at month 1 +5.6 cm at month 6	Y	U
Rumbo et al. (17)	6 days/week	U	0 ml/kg/day at week 25	1 at week 25	U	Improved (weaned)	Improved BMI/A 1.32 at start, 0.54 at week 50)	Improved H/A-3.66 at start, −2.51 at week 50)	Y	U
Sigalet et al. (9)^z^	66% of calories intake ± 16	34% of calories intake ± 16	54% of calories intake	0	25% ± 18 of calories intake	75% ± 46 of calories intake	Baseline: 4.7 Kg ± 1.6 42 days: 5.3 kg ±1.2	U	Y	Baseline: 7.8 ± 1.7 42 days: 10.5 ± 3.4 μmol/L
Sigalet et al. (12)	U	U	Stable	0	Stable	Stable	Increased	Increased	Y	Increased
Mercer et al. (21)^z^	U	U	−42.4% ± 29.19 at C1D1 (*n* = 33), and −49.6% ± 32.57 at C1W24 (*n* = 25), −1.0 ± 1.89 days per week at C1D1 (*n* = 33) and −1.4 ± 2.62 days per week at C1W24 (*n* = 25).	7 at C1W24	U	U	Stable	Stable	U	U
Total				36						

*PN, Parenteral Nutrition; EN, Enteral Nutrition; U, Unknown; PN/REEI, PN intake in calories/resting energy expenditure.*

*^x^Median (range), ^y^Mean (range), ^z^Mean ± Standard Deviation.*

In 7 studies (9, 12, 15, 17, 19, 20, 25), patients were evaluated for changes in weight or height with an increase in weight in 6 cases and height in 5. However, in one study (25), patients did not improve weight or height after treatment.

Eight studies out of 14 evaluated changes in stool’s quality and all of them showed an improvement in patients’ stool either in reducing the number of evacuations per day or in better quality of stool (9, 12, 15–17, 19, 20, 25).

Seven studies evaluated plasmatic levels of citrulline, showing higher plasmatic levels if compared to baseline (9, 12, 16, 18, 20, 24, 25).

Eleven studies reported complications on treatment: gastrointestinal complications were the most common event, with 87 cases reported in treated patients and 10 in non-treated patients. Other complications (more than two events) were upper respiratory tract infections, CVC related complications, pyrexia, hepatobiliary or pancreatic disorder, dehydration, or electrolyte dysfunctions or injections site bruises. One study reported insurgence of abdominal distensions, loss of appetite, and worsening diarrhea in one patient after 2 weeks of treatment discontinuation due to unexpected drug supply issues (15). The most common complications are listed in Table 6.

**TABLE 6:** Adverse events emerged during treatment period; different complications may have developed in the same patient.

References	Gastrointestinal; treated patients, SOC	Upper respiratory tract infection; treated patients, SOC	CVC related complication; treated patients, SOC	Pyrexia; treated patients, SOC	Sepsis; treated patients, SOC	Hepatobiliary/ pancreatic; treated patients, SOC	Electrolyte alterations; treated patients, SOC	Dehydration; treated patients, SOC	Cardiac decompensation; treated patients, SOC	Injection site complications; treated patients, SOC	Urinary complications; treated patients, SOC
Carter et al. (18)^vw^	21, 3	19, 3	13, 1	9, 2	–	–	–	–	–	3	–
Busoni et al. (19)	–	–	–	–	1	1	–	–	–	–	–
Ferreiro et al. (20)	–	–	–	–	–	–	–	–	–	–	–
Hill et al. (22)^x^	6	6	–	–	–	–	–	–	–	–	–
Kinberg (23)^y^	–	–	–	–	–	1	4	–	–	–	–
Lambe (25)^z^	1	–	2	2	–	–	–	1	–	–	–
Kocoshis et al. (24)	30, 7	15, 4	4	19, 4	–	5	–	9	–	4	3, 1
Martìnez et al. (26)	2	1	–	–	–	–	–	–	–	1	1
Ramos-Boluda et al. (16)^z^	1	–	–	–	–	1	–	–	1*	–	–
Ribeiro-Mourão et al. (15)	4	1	–	–	–	–	–	–	–	–	–
Rumbo et al. (17)	–	–	–	–	–	–	–	–	–	–	–
Sigalet et al. (9)	–	–	–	–	–	–	–	–	–	–	–
Sigalet et al. (12)^v^	2	–	–	–	–	–	–	–	–	–	–
Mercer et al. (21)	20	–	–	1	–	8	–	–	–	2	–
Total	87, 10	42, 7	19, 1	31, 6	1	16	4	10	1	10	4, 1

*Due to intercurrent disease; ^v^Adverse events were described as not related to Tedugliutide; ^w^Complications were described when occurring in > 5% of patients. ^x^Adverse events were reported by 93.8% of patients, only 2 events were considered related to Teduglutide**;** ^y^Gastrointestinal complications were the most common event, but the paper did not include data; ^z^Abdominal pain, stoma changes, redness at the injection site and legs and muscle pain are reported without specific data. SOC, Standard of Care; CVC, Central Vein Catheter.

## Discussion

SBS is a complex condition that affects around 24.5 children every 100,000 live births (2, 18). Achieving EA is the crucial goal in treating these patients to improve their quality of life, reduce complications and prolong their life expectancy.

In the last 40 years, treatment of SBS has changed dramatically since the introduction of Autologous Gastrointestinal Reconstruction procedures (AGIR) pioneered by Adrian Bianchi in the ‘80s (27). Non-transplant surgery establishes itself as therapy when dealing with SBS (28). Furthermore, AGIR procedures, when performed as part of multidisciplinary treatment, help reduce the need for PN and achieve EA (29). Therefore, it is crucial to find a holistic approach (medical and surgical) to treat this rare and complex condition.

Teduglutide was approved in 2016 to treat patients older than 1 year of age affected by IF. It has proven to be safe in this population and adults, and the studies included in our review confirm the safety of the treatment. Side effects and possible adverse reactions reported in the drug leaflet are respiratory tract infections, anxiety and insomnia, headache, gastrointestinal disorders, congestive heart failure, hepatobiliary, and pancreatic disorders, injections site reactions and stoma complications. The findings in our study are primarily consistent with what was already known of adverse effects based on adults’ studies. At the same time, anxiety and insomnia were not reported as typical (more than two events) in any of the studies included.

This study aimed to evaluate the efficacy of Teduglutide in reducing the need for PN in children affected by SBS-IF. Six studies among inclusion criteria specified that patients should be stable at baseline with no changes in PN support in the last 3 months at least (15, 16, 18, 19, 24, 25). Two studies only included patients at least 45 days apart from previous intestinal surgery (9, 12). It is essential to understand that weaning patients from PN could mean reducing their risk of Catheter-Associated Bloodstream Infections (CABSI) dramatically, preventing IF Associated Liver Disease (IFALD) and thrombosis, without mentioning the quality-of-life improvement and reduction of costs for the national health system (30, 31). Among included studies 16% of patients achieved EA, and 67% showed a decrease in PN needs in terms of volume, calories, or hours per day after the trial period of treatment. All studies included showed at least a partial reduction in PN requirements and demonstrated the safety of Teduglutide in the pediatric population. One study included infants treated with doses of 0.005–0.02 mg/kg/day in divided doses with no adverse events correlated to GLP-2 treatment (9).

Seven studies included showed augmentation of plasmatic Citrulline levels along with Teduglutide treatment. Citrulline can be used as a marker of intestinal function in situations where there is a significant loss of enterocytes’ mass and function, its levels appear to be strongly correlated with small bowel length and intestinal absorption in patients affected by SBS (32, 33). The correlation between citrulline levels and enteral absorption appears to be moderate, but it is strongly correlated with intestinal length and state. Citrulline levels appear higher in patients treated with Teduglutide, and this finding is consistent with our analysis (33).

Patients suffering from IF still have a high mortality rate that ranges from 30 to 50% (28). Survival of these patients also depends on comorbidities and complications due to their condition as they are exposed to Catheter Induced Bloodstream Infections, IF Associated Liver Disease, thrombosis, electrolyte dysfunction, and malnutrition. In the last years, survival has improved after the introduction of multidisciplinary intestinal rehabilitation programs, autologous bowel-lengthening procedures, and the use of new and improved formulas in PN (34). To improve their chances of survival and intestinal function, we should promptly refer these patients to highly specialized centers to get a multidisciplinary treatment that involves different professional medical and surgical treatment. Treatment in SBS-IF should not be considered a single process, and patients should be included in a personalized program that involves both medical and surgical approaches. AGIR is a fundamental part of SBS-IF treatment as it allows to gain intestinal lengths and maximize its function by improving transit and reducing intestinal dilation. AGIR procedures consist of a systematic and personalized approach to SBS, and it should be planned as part of an IRP program. Among available medical treatments in the pediatric population, Teduglutide appears safe and effective. It should be considered part of the tools in the hand of professionals to help these patients.

This review has different limitations that should be recognized. Data are extracted from both prospective and retrospective studies, and we included clinical trials and case reports. Only a limited number of studies was available at this systematic review, and only 14 studies completely fulfilled the inclusion criteria. Given the paucity of records, we decided to include abstracts without a full article available: this could impact the overall quality of included studies. Limitations concerning the availability of data and information were previously reported in the quality assessment in the results section: small sample size and short follow up (9, 12, 15, 18, 19, 26), different periods of treatment with teduglutide (26) and absence of stratification of results based on age bowel length (16, 23) and teduglutide dose (22).

In many studies, we did not find the intestinal size, previous surgery or height and weight before and after treatment. In some cases, PN and EN regimens before or after treatment were not described. Another limiting factor is the different doses at which Teduglutide was administered; the exact amount was also not specified in three papers (9, 15, 23). Finally, we must acknowledge the diversity in included studies regarding measurements of primary and secondary outcomes; data were not expressed uniformly in different papers: some used median, and range and others used mean and range or standard deviation, PN requirements were defined in ml/kg/day as well as the percentage of total intake, weight and height were reported in Kg and cm as well as Z-score. A standard definition of primary outcome across all the Intestinal Rehabilitation Units would be beneficial to measure across different units how GLP-2 can effectively help SBS patients.

Despite limiting factors, our work could contribute to the scientific community: this is the first systematic review ever conducted on the use of Teduglutide in the pediatric population. SBS is a rare disease that has a high impact on the lives of affected patients and their families. It is fundamental to give these children more options for treatment possibilities. Teduglutide seems to be a promising opportunity in selected SBS patients that must be considered when thinking of a multidisciplinary approach to this complex condition. It should be proposed for treatment in patients over 1 year of age affected by SBS who are stable following a period of intestinal adaptation after surgery. The recommended dose is 0.05 mg/kg body weight once daily.

Since its approval in 2016, different studies have been conducted on the pediatric population, but more data on the long-term efficacy of Teduglutide and complications related to treatment are needed. Moreover, it still must be studied and understood if the improvement in achieving EA and reducing PN requirements persists in the long-term, especially after treatment discontinuation. Only four studies out of the 14 included in this systematic review analyzed the PN requirement after treatment discontinuation (9, 12, 18, 24). Two studies showed an increase in PN requirements, one showed no change, and one showed a decrease in the percentage of calories taken enterally at 1 month after discontinuation (without going back to pre-treatment needs) and an increase in EA at 6 and 12 months after treatment.

## Conclusion

This study represents the first systematic review on the efficacy of Teduglutide in the pediatric population. Our work shows that teduglutide appears safe in treating patients under 18 years of age and effectively reduces PN requirements and improves EA in pediatric patients affected by SBS. Teduglutide is a valuable tool in the hands of professionals who treat these patients, and it should be considered when creating a multidisciplinary treatment plan for these children. However, more studies are needed to fully understand the complications related to long term treatment and the efficacy of Teduglutide, especially after discontinuation of treatment.

## Data Availability Statement

The original contributions presented in this study are included in the article/supplementary material, further inquiries can be directed to the corresponding author.

## Author Contributions

FG, RCi, RCo, and AM contributed to conception and design of the study. FG and RCi organized the database. FG, RCi, and MDR performed data extraction. MCC and MCR performed the quality assessment. FG wrote the first draft of the manuscript. MCR, RCo, RCi, MCC, MDR, and AM wrote sections of the manuscript. All authors contributed to manuscript revision, read, and approved the submitted version.

## Conflict of Interest

The authors declare that the research was conducted in the absence of any commercial or financial relationships that could be construed as a potential conflict of interest.

## Publisher’s Note

All claims expressed in this article are solely those of the authors and do not necessarily represent those of their affiliated organizations, or those of the publisher, the editors and the reviewers. Any product that may be evaluated in this article, or claim that may be made by its manufacturer, is not guaranteed or endorsed by the publisher.

## References

[1] Nagelkerke SCJ van Oers HA Haverman L Vlug LE de Koning BAE Benninga MA Longitudinal development of health-related quality of life and fatigue in children on home parenteral nutrition. *J Pediatr Gastroenterol Nutr.* (2022) 74:116–22. 10.1097/MPG.0000000000003329 34694264 PMC8673843

[2] Muto M Kaji T Onishi S Yano K Yamada W Ieiri S. An overview of the current management of short-bowel syndrome in pediatric patients. *Surg Today.* (2022) 52:12–21. 10.1007/s00595-020-02207-z 33464414

[3] Struijs M-C Diamond IR de Silva N Wales PW. Establishing norms for intestinal length in children. *J Pediatr Surg.* (2009) 44:933–8. 10.1016/j.jpedsurg.2009.01.031 19433173

[4] Coletta R Aldeiri B Morabito A. Institutional experience with spiral intestinal lengthening and tailoring. *Eur J Pediatr Surg.* (2018) 29:412–6. 10.1055/s-0038-1660850 29920633

[5] Quirós-Tejeira RE Ament ME Reyen L Herzog F Merjanian M Olivares-Serrano N Long-term parenteral nutritional support and intestinal adaptation in children with short bowel syndrome: a 25-year experience. *J Pediatr.* (2004) 145:157–63. 10.1016/j.jpeds.2004.02.030 15289760

[6] Bianchi A Morabito A. The dilated bowel: a liability and an asset. *Semin Pediatr Surg.* (2009) 18:249–57. 10.1053/j.sempedsurg.2009.07.010 19782307

[7] Bremholm L Hornum M Henriksen BM Larsen S Holst JJ. Glucagon-like peptide-2 increases mesenteric blood flow in humans. *Scand J Gastroenterol.* (2009) 44:314–9. 10.1080/00365520802538195 19005872

[8] Brubaker PL Anini Y. Direct and indirect mechanisms regulating secretion of glucagon-like peptide-1 and glucagon-like peptide-2. *Can J Physiol Pharmacol.* (2003) 81:1005–12. 10.1139/y03-107 14719035

[9] Sigalet DL Brindle ME Boctor D Dicken B Lam V Lu LS A safety and pharmacokinetic dosing study of glucagon-like peptide 2 in infants with intestinal failure. *J Pediatr Surg.* (2017) 52:749–54. 10.1016/j.jpedsurg.2017.01.034 28209419

[10] Thulesen J. Glucagon-like peptide 2 (GLP-2), an intestinotrophic mediator. *Curr Protein Pept Sci.* (2004) 5:51–65. 10.2174/1389203043486946 14965320

[11] Jeppesen PB. Teduglutide (ALX-0600), a dipeptidyl peptidase IV resistant glucagon-like peptide 2 analogue, improves intestinal function in short bowel syndrome patients. *Gut.* (2005) 54:1224–31. 10.1136/gut.2004.061440 16099790 PMC1774653

[12] Sigalet DL Brindle M Boctor D Casey L Dicken B Butterworth S Safety and dosing study of glucagon-like peptide 2 in children with intestinal failure. *J Parenter Enteral Nutr.* (2015) 41:844–52. 10.1177/0148607115609566 26471991

[13] Page MJ McKenzie JE Bossuyt PM Boutron I Hoffmann TC Mulrow CD The PRISMA 2020 statement: an updated guideline for reporting systematic reviews. *BMJ.* (2021) 372:n71. 10.1136/bmj.n71 33782057 PMC8005924

[14] Sterne JA Hernán MA Reeves BC Savović J Berkman ND Viswanathan M ROBINS-I: a tool for assessing risk of bias in non-randomised studies of interventions. *BMJ.* (2016) 355:i4919. 10.1136/bmj.i4919 27733354 PMC5062054

[15] Ribeiro-Mourão F de Braganca RL Nogueira M Guerra P Espinheira C Trindade E Short-term results of teduglutide therapy in children with short bowel syndrome. *J Pediatr Gastroenterol Nutr.* (2021) 72:1300. 10.1016/j.jpeds.2016.10.027 27855998

[16] Ramos Boluda E Redecillas Ferreiro S Manrique Moral O García Romero R Irastorza Terradillos I Nuñez Ramos R Experience with teduglutide in pediatric short bowel syndrome: first real-life data. *J Pediatr Gastroenterol Nutr.* (2020) 71:734–9. 10.1097/MPG.0000000000002899 32804906

[17] Rumbo C Martinez MI Gondolesi GE Fernandez A. Intestinal rehabilitation in Latin-America, report of the first paediatric case treated with Teduglutide, fifty weeks of follow up. *J Pediatr Gastroenterol Nutr.* (2018) 66:391–2.28837513

[18] Carter BA Cohran VC Cole CR Corkins MR Dimmitt RA Duggan C Outcomes from a 12-week, open-label, multicenter clinical trial of teduglutide in pediatric short bowel syndrome. *J Pediatr.* (2017) 181:102–11.e5. 27855998 10.1016/j.jpeds.2016.10.027

[19] Busoni V Izquierdo C Frangi F Lobos P Orsi M. Initial experience with teduglutide in pediatric intestinal failure. *J Pediatr Gastroenterol Nutr.* (2021) 72:1284.

[20] Ferreiro SR Ruiz VC Martínez LG Ramos RN Recio JB Canton S. *Teduglutide?: The New Weapon in Pediatric Short Bowel Syndrome Pediatric Gastroenterology, Hepatology and Nutrition Unit.* Barcelona: Hospital Vall d’ Hebrón. (2019).

[21] Mercer D Carter B Hill S Horslen S Kaufman S Kocoshis S A prospective, open-label, long-term safety and efficacy study of teduglutide in pediatric patients with short bowel syndrome-associated intestinal failure: 6-month interim analysis. *J Pediatr Gastroenterol Nutr.* (2019) 69:11–4.

[22] Hill S Carter B Horslen S Kocoshis S Yoon MJ Grimm A. A prospective, open-label, long-term safety and efficacy study of teduglutide in pediatric patients with short bowel syndrome-associated intestinal failure: 6-month interim analysis (SHP633-303). *J Pediatr Gastroenterol Nutr.* (2019) 69:2019–22.

[23] Kinberg S. Single-center experience with teduglutide in pediatric patients with short bowel syndrome associated intestinal failure. *Transplantation.* (2021) 105:S67–67. 10.1097/01.tp.0000757952.00163.8c34908286

[24] Kocoshis S Carter BA Hill S Horslen S Li B Goyal S Intestinal adaptation in children with short bowel syndrome during treatment with teduglutide. *J Parente Enteral Nutr.* (2016) 40:132–3.

[25] Lambe CA. Monocentric single-arm study on long-term safety and efficacy of teduglutide in SBS pediatric patients on long-term home-parenteral nutrition. *Transplantation.* (2021) 105:S2–2. 10.1097/01.tp.0000757480.19792.d735704014

[26] Martinez MI Rumbo C Fernández A Ramisch D Gondolesi GE. Teduglutide: intestinal rehabilitation in children, our initial experience. *Transplantation.* (2019) 103:PS162.

[27] Bianchi A. Intestinal loop lengthening—a technique for increasing small intestinal length. *J Pediatr Surg.* (1980) 15:145–51. 10.1016/S0022-3468(80)80005-4 7373489

[28] Massironi S Cavalcoli F Rausa E Invernizzi P Braga M Vecchi M. Understanding short bowel syndrome: current status and future perspectives. *Dig Liver Dis.* (2020) 52:253–61. 10.1016/j.dld.2019.11.013 31892505

[29] Coletta R Morabito A. Non-transplant surgical management of short bowel syndrome in children: an overview. *Curr Pediatr Rev.* (2019) 15:106–10. 10.2174/1573396315666181129164112 30499416

[30] Spencer AU Kovacevich D McKinney-Barnett M Hair D Canham J Maksym C Pediatric short-bowel syndrome: the cost of comprehensive care. *Am J Clin Nutr.* (2008) 88:1552–9. 10.3945/ajcn.2008.26007 19064515

[31] Fonseca G Burgermaster M Larson E Seres DS. The relationship between parenteral nutrition and central line-associated bloodstream infections: 2009-2014. *J Parenter Enteral Nutr.* (2018) 42:171–5. 10.1177/0148607116688437 29505142 PMC5568511

[32] Crenn P Messing B Cynober L. Citrulline as a biomarker of intestinal failure due to enterocyte mass reduction. *Clin Nutr.* (2008) 27:328–39. 10.1016/j.clnu.2008.02.005 18440672

[33] Fragkos KC Forbes A. Citrulline as a marker of intestinal function and absorption in clinical settings: a systematic review and meta-analysis. *United Eur Gastroenterol J.* (2018) 6:181–91. 10.1177/2050640617737632 29511548 PMC5833233

[34] Gattini D Roberts AJ Wales PW Beath SV Evans HM Hind J Trends in pediatric intestinal failure: a multicenter, multinational study. *J Pediatr.* (2021) 237:16–23.e4. 10.1016/j.jpeds.2021.06.025 34153281

